# High-Throughput Prediction of Whole Season Green Area Index in Winter Wheat With an Airborne Multispectral Sensor

**DOI:** 10.3389/fpls.2019.01798

**Published:** 2020-02-14

**Authors:** Josephine Bukowiecki, Till Rose, Ralph Ehlers, Henning Kage

**Affiliations:** Institute of Crop Science and Plant Breeding, Christian-Albrechts-University, Kiel, Germany

**Keywords:** green area index, unmanned aerial vehicle, multispectral, winter wheat, whole season, vegetation index, sequoia camera

## Abstract

**Introduction:**

In recent decades, the interest has grown to quantify the green area index as one of the key characteristics of crop canopies (e.g. for modelling transpiration, light interception, growth). The approach of estimating green area index based on multispectral reflection data from unmanned airborne vehicles with lightweight sensors might have the potential to deliver data with sufficient accuracy and high throughput during the whole season.

**Materials and Methods:**

We therefore examined the applicability of a recently launched drone-based multispectral system (Sequoia, Parrot) for the prediction of whole season green area index in winter wheat, with data from field trials in Northern Germany (2017, 2018 and 2019). The explanatory power of different modeling approaches to predict green area index based on multispectral data was tested: linear and non-linear regression models, multivariate techniques, and machine learning algorithms. Further, different predictors were implemented in these models: multispectral data as raw bands and as ratios. Additionally, a new approach for the evaluation of green area index predictions during senescence is introduced. It is shown that a robust calibration during growth phase is applicable during senescence as well.

**Results and Discussion:**

A linear model which includes all four wavebands provided by the sensor in three ratios (VIQUO) and a Support Vector Machine (SVM) algorithm allow a reliable and sufficiently accurate whole season prediction. The VIQUO-model is recommended as the best model, as it is precise but still relatively simple, thus easier to communicate and to apply than the SVM. The integrated values of predicted green area indices in an independent trial are highly correlated with their final biomass (R^2^: VIQUO = 0.84, SVM = 0.85) which represents the process of radiation interception, one of the determining factors of growths. This is an indicator for both, a robust model calibration and a high potential of the tested multispectral system for agricultural research and crop management.

## Introduction

In agricultural science, the monitoring of canopies throughout the growing season is of major concern to understand, predict, and manage crop growth. A fundamental parameter of canopies is the green area index (green plant area per ground area; *GAI*), which plays a central role in the processes of radiation interception and transpiration by vegetation. Because *GAI* changes continuously from sowing to harvest, sequenced measurements are necessary. For this purpose, non-destructive methods are advantageous. Additionally, applications in precision agriculture and the phenotyping of large plant material collections require the measurement of large areas in feasible time. Both demands are fulfilled by remote spectral sensors (Salamí et al., 2014).

Since the first introduction of remote sensing data for the satellite driven surveillance of vegetated areas, this field of research has developed significantly (Weiss et al., 2020). In the same time, new scopes of application of spatial data have emerged. It became quickly evident that satellite sensors cannot meet all requirements of remote sensing applications, due to insufficient spatial and temporal resolution. To overcome these restrictions, new platforms for complemental or alternative data acquisition were introduced (e.g. onboard traction-engine, airplanes) (Zecha et al., 2013).

First developed for military applications (Majumdar et al., 2001; Beard et al., 2006), unmanned aerial vehicles (UAV) turned in focus of civilian remote sensing with special attention in the domain of agriculture (Salamí et al., 2014). With their measurement distance between ground (tractor, handheld) and satellite, UAV can reach both, high ground coverage as well as high spatial resolution. Furthermore, they are independent of cloud cover (in contrast to satellites) and are in some environments the only opportunity to reach sufficient temporal resolution within certain time periods during season. Considering these features, UAVs might be the most promising carrier systems for airborne spectral sensors in agricultural research and precision agriculture (Puri et al., 2017; Raparelli and Bajocco, 2019; Tsouros et al., 2019).

Despite the high spatial resolution of UAV-data, there are still a lot of factors influencing the spectral reflectance signal, such as soil background and radiation conditions. To compensate this spectral variability, it is common practice to associate crop characteristics not only with one spectral band, but with at least two, merged into a vegetation index (VI) (Tucker et al., 1981; Baret and Guyot, 1991; Verger et al., 2014).

In recent decades, a high number of VIs have been developed to characterize different crop characteristics. The certainly most popular VI is the normalized difference vegetation index (NDVI) by Rouse et al. (1974), combining the spectral range of red with the near infrared (NIR). The NDVI has been shown to be sensitive to different crop characteristics, such as leaf area index (*LAI*), *GAI*, dry matter, and nitrogen content (e.g. Serrano et al., 2000; Lelong et al., 2008; Berni et al., 2009; Mistele and Schmidhalter, 2010; Nebiker et al., 2016; Ni et al., 2017). However, it is a known fact that the sensitivity of the NDVI towards these crop characteristics is heavily dependent on the degree of soil coverage, being insensitive above a *LAI* of 2–3 m^2^ m^-2^ (Serrano et al., 2000; Haboudane et al., 2004; Pinty et al., 2009; Viña et al., 2011). Thus, for the most crops the NDVI cannot provide information for a long time in the vegetation period. Several newly developed VIs are more or less able to compensate this saturation effect, but are still subject to a number of influences restricting their usability through the whole growing season and on different sites, such as different species, growth stages, the process of senescence, site- and year-effects (e.g. Gitelson et al., 2003; Serrano et al., 2000; Li et al., 2010).

In recent time, new, multivariate methods as well as non-linear algorithms have been introduced in the calibration of spectral data to crop data, such as Partial Least Squares Regression (Höskuldsson, 1988) or Support Vector Machines (SVM) (Burges, 1998). As a consequence of this broad field of different VIs, used spectral bands, different multispectral sensors, and different prediction models, the best way to use a spectral sensor for the prediction of any canopy characteristic might be reassessed separately for each new sensor model.

Therefore, the objective of this study was to develop an easy-to-handle, reliable UAV-based approach to predict whole season *GAI* of winter wheat which could usefully be transferred into precision agriculture and support agricultural research. For this purpose, a recently launched low to medium cost UAV-based multispectral system was deployed, namely the Parrot Sequoia sensor.

The focus was on the questions: (1) Is the Sequoia sensor providing sufficiently meaningful multispectral images for the monitoring of winter wheat growth on plot level? (2) How can the data be used best for the prediction of the crop characteristic *GAI*? (2a) Can one calibration approach for *GAI* be employed throughout the whole growing season? (2b) Are the VI-based approaches excelled by the new multivariate methods?

## Materials and Methods

### Study Site and Trial Design

Data acquisition was conducted during three years (growing seasons 2016/17, 2017/18 and 2018/19) at the Hohenschulen Experimental Farm (10.0 E, 54.3 N, 30 m a.s.l.) of the Kiel University, located in Northern Germany. The long-term average temperature is 8.9°C, the precipitation average 788 mm (Deutscher Wetterdienst, 2013). The site is characterized by a small-scale heterogeneous soil, the main soil type being a pseudogleyic sandy loam.

Destructive sampling for sensor calibration was conducted as additional measurement in different ongoing trials. In the growing season 2016/17, data were collected within Trial A and Trial B, in 2017/18 in Trial B and Trial C and in 2018/19 only in Trial C. Trial A is a four-field rotation since 2003, with winter wheat following winter oilseed rape. Four different nitrogen levels are tested in interaction with four different cultivars in four replications (**Table 1**). Trial B and C are experimental sites, placed each year on a different field. In Trial B four different sowing densities of four cultivars are examined whereas in Trial C six cultivars and two nitrogen levels are tested (**Table 1**). Except the treatments mentioned, the wheat crops were managed according to regional farmer’s practice.

**Table 1:** Different treatments of the three field trials.

Trial (Replications)	Treatments		
	Cultivar	Nitrogen levels and split application rates [kg N ha^-1^]	Sowing Seed Density [Kernels m^-2^]
Trial A (n = 3)	Benchmark Dekan KWS Maddox RGT Reform	0: 0/0/0 80: 40/40/0 160: 80/40/40 240: 80/80/80	270
Trial B (n = 3)	Brilliant Dekan Piko Solehio	200: 80/80/60	50 100 200 400
Trial C (n = 4)	Elixer Hybery JB Asano Piko SUR99820 Solehio	110: 50/60/0 220: 50/110/60	280

### Data Collection

**Figure 1:**
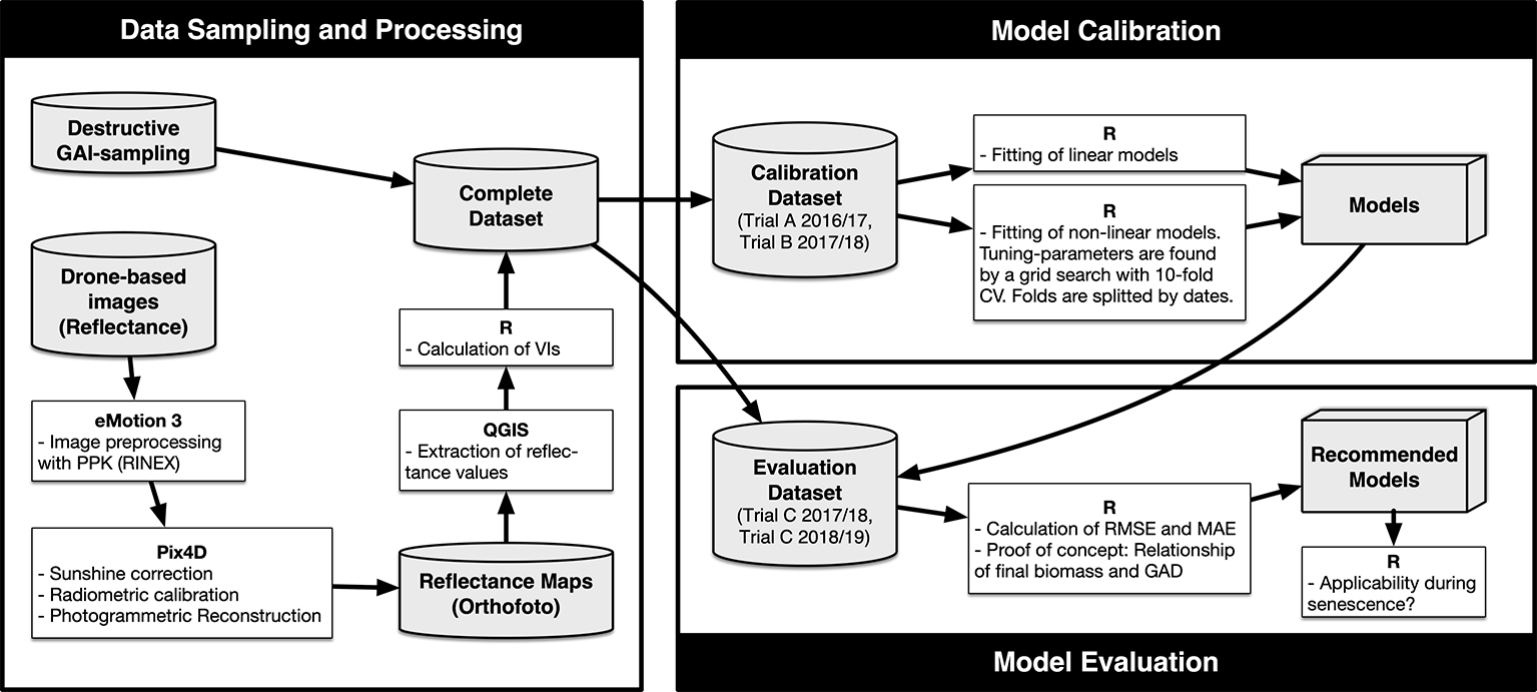
Flowchart of the process of data collection, data processing, model calibration, and model evaluation (CV, cross validation; GAD, green area duration; MAE, mean absolute error; RMSE, root mean square error; VI, vegetation index).

The following sections (*GAI Reference Measurement* up to *Advanced Predictive Models*) describe the process from data collection through data processing to model calibration and evaluation. To clarify the procedure, it is illustrated schematically in **Figure 1**.

#### GAI Reference Measurement

At every sampling date (**Table 2**), three replications of the respective trial were sampled, hence 48 plots. In every plot, the aboveground plant material of an area of 0.25 m² was withdrawn and its BBCH stage (Lancashire et al., 1991) was scored. The fresh matter was fractionized into leaf, stem, ear, and senescence. The category senescence comprised all fresh matter no longer considered as green and was the only fraction not being included in the calculation of the *GAI*, wherefore the *LAI*, Stem Area Index and Ear Area Index were determined using a LiCor 3100 leaf area meter (LiCor Inc., NE, USA).

**Table 2:** Sampling dates and the most frequent BBCH stage (mode).

Trial	Sampling date (BBCH)		
	Season 2016/17	Season 2017/18	Season 2018/19
Trial A	2017-04-11 (31) 2017-05-16 (37) 2017-06-07 (65) 2017-06-27 (75) 2017-08-07 (92) 2017-08-29 (0)	–	–
Trial B	2017-04-04 (30) 2017-05-09 (33) 2017-05-30 (51) 2017-07-18 (83)	2018-03-14 (21) 2018-04-24 (29) 2018-05-22 (37)	–
Trial C	–	2018-04-18 (24) 2018-05-15 (37) 2018-06-04 (61) 2018-07-17 (92)	2018-11-27 (21) 2019-04-09 (31) 2019-05-06 (33) 2019-06-03 (55)

On August 07, 2017 and on July 17, 2018 ripening was considered as terminated (BBCH 92), thus, at these dates a *GAI* of 0 was assigned to each plot of the relevant trial. Spectral data from the bare ground of the plots from Trial A at August 29, 2017 were included in the dataset as well.

A description of the *GAI*-course during the senescence by destructive sampling and measurements of the green area is not possible as the differentiation between green and senesced plant material is not clearly defined and because, in parallel to this zonal process, a gradual degradation of chlorophyll takes place. The concept to evaluate *GAI*-predictions during this development stage is further specified in *Further Evaluation of GAI-Models*.

#### Reflectance Measurements

Every sampling date was accompanied by an overflight with an UAV-based multispectral camera within at most three days. The plant development during this time span was considered as negligible, thus no interpolation techniques (as for example used by Broge and Mortensen, 2002) were applied. An overflight after the sampling was in each case used to know at which position the samples were taken exactly, by visual determination of the additional hole in the canopy. For further evaluation of the *GAI*-models, 14 additional overflights on Trial C in the season 2017/18 were made (April 04, April 16, April 23, May 03, May 16, May 22, June 01, June 06, June 12, June 20, June 26, July 05, July 13, July 17).

The UAV applied was an eBee by senseFly, a micro aerial vehicle (following the classification of Allen et al., 2011), with fixed wings and automatic flight manager. It served as carrier system for the Parrot Sequoia camera (Parrot Drones SAS, France, Paris), a multispectral sensor which records simultaneously images in four reflection-bands; green (550 nm), red (660 nm), red edge (RE, 735 nm), and near-infrared (NIR, 790 nm). Besides RE, all reflection-bands have a bandwidth of 40 nm, RE just of 10 nm. The Sequoia camera has an incoming light sensor and provides therefore fractional reflection values regarding the incoming radiation. Before each flight, images of a grayscale target were made for radiometric calibration.

The software eMotion3 from senseFly was used as flight manager. Every position on the ground was at least photographed five times to ensure a sufficient data quality. The chosen resolution was 8 x 8 cm pixel^-1^. All images were preprocessed in the post flight-manager of eMotion 3 and afterwards imported and processed using the Pix4Dmapper software (*Pix4D* SA., Switzerland). On days with fast moving clouds, a manual screening of the images was conducted to exclude those images containing both, regions with cloud shadow and full sunlight. The results were four orthogonal reflection maps, one for every waveband. The extraction of the reflectance data of the sampling spots was undertaken in QGIS version 3.8.0 (QGIS Development Team, 2018), whereby all pixels of a sampling spot were summarized as median.

With a RTK-enabled eBee, it is possible to include RINEX-files (Receiver Independent Exchange Format) in the post-flight-processing in eMotion 3. If this function was not available, the reflectance maps were georeferenced manually using the Georeferencer Plugin in QGIS.

## Model Calibration

### Data Sets

At the very beginning, plots assessed as compromised in any manner (e.g. strong weed abundance, damages by game, lodging) were excluded of further consideration. This affected especially Trial B, as in 2017 the data of the whole sowing density 50 K m^-2^-treatment were sorted out due to strong weed abundance and the data of sowing density 400 K m^-2^ of the cultivar Solehio after early July due to lodging. In 2018 in Trial B, problems in crop establishment of the cultivar Dekan and additional waterlogging stress led to too little sampling material in the lowest sowing density variant at the end of May and in the two lowest sowing density variants in June.

Considering *GAI*, values above 7 m² m^-2^ were excluded from the dataset because under local growing conditions these high values do never occur as a treatment mean and they were considered as more harmful then useful for calibration purposes (concerns six samples of the calibration dataset and one of the evaluation dataset). Furthermore July 18, 2017; June 27, 2017 and June 11, 2018 were omitted from *GAI*-dataset as the process of senescence had already started (see *GAI Reference Measurement*).

Finally, the data from Trial A and Trial B (2016/17 and 2017/18) were combined to form one single calibration dataset, covering a broad spectrum of *GAI*-values, crop managements, and environmental factors (e.g. irradiance at flight day, ground coverage, nitrogen levels and sowing densities), whereas the data from Trial C constitute the evaluation dataset (45% of the total data volume). Calibration and evaluation dataset never share a common date and flight, respectively. The result is a high level of independence between both data sets (final sample size for *GAI*-calibration: 474, *GAI*-evaluation: 383).

### Statistical Analysis

All statistical analysis was conducted in R Core Team, 2017.

#### Linear Regression Models

Every single band, their combination with and without interaction, the quotients of NIR and Green, NIR and Red, NIR and RE, their combination with and without interaction and the NDVI (the quotient of NIR-Red and NIR+Red, by Rouse et al., 1974), as most common VI, were tested for their sensitivity towards *GAI*.

Based on the calibration dataset, linear models between *GAI* and the components of the VIs were fitted, whereas for the NDVI an exponential term was introduced, taking into consideration its known non-linear behavior (Wiegand et al., 1992; Chen and Cihlar, 1996; Myneni et al., 1997; Lelong et al., 2008; Viña et al., 2011).

For the comparison of the different VIs, two statistical metrics were selected: the root mean square error (RMSE) and the mean absolute error (MAE). The RMSE was chosen for comparability with the results of other studies. However, since the RMSE gives more weight to large errors but the predictive power of the models at low *GAI*-values is at least as important as at high *GAI*-values (non-linear relationship between light interception and *GAI*), the MAE was used to compare the advantages and disadvantages of the different models.

#### Advanced Predictive Models

To streamline the process of model creation, the integrative package *caret* (Kuhn, 2017) was used. The models were implemented by different additional packages: Partial Least Squares Regressions using *pls* (Mevik et al., 2016), SVM using *kernlab* (Karatzoglou et al., 2004), K Nearest Neighbor using *caret* (Kuhn, 2017), Multivariate Adaptive Regression Spline using *earth* (Milborrow, 2017), and Boosted Trees using *gbm* (Ridgeway, 2017).

For Partial Least Squares, SVM, and K Nearest Neighbor, all predictors were centered and scaled prior to model fitting. The models Partial Least Squares, SVM, K Nearest Neighbor, Multivariate Adaptive Regression Spline, and Boosted Trees possess tuning parameters. These were found by a grid search and the optimal model was selected by the smallest RMSE value. The parameters usually have a tradeoff between descriptive and predictive modeling performance. To prevent overfitting by an optimization of the descriptive quality, the RMSE for parameter selection was calculated by a 10-fold cross-validation. The hold-out sample was specifically selected by date. Tuning parameter “n.minobsinnode” for the Boosted Tree model was held constant at the value of 10. Selected tuning parameters are shown in **Table S1**.

#### Further Evaluation of GAI-Models

To examine the sensitivity of some selected VIs through the growing season, the dataset was further divided in seven different *GAI*-classes and the MAE and the relative MAE (rMAE) of the *GAI*-predictions in the different *GAI*-classes was calculated individually. As the rMAE is determined as the quotient of the MAE and the mean *GAI* of the considered class, no rMAE could be calculated for the class “Dead Plant” (division through 0 not possible).

Further it was considered if one *GAI*-model can be applied regardless of the cultivar (unaffected by different leaf angles and single leaf reflectance). For this purpose, calibration and evaluation dataset were reduced to the data of the two cultivars represented in both datasets: Solehio and Piko. Those two cultivars are quite contrasting ones (**Table 3**): Piko represents a compact growth habitus with rather short and planophile leaves while Solehio shows a pronounced vertical growth with long, erectophile leaves. Based on the calibration subset, two linear models were calibrated; a “common” model which estimates the *GAI via* reflectance data only and an “extended” model, including the cultivar as an additional factor. The effect of cultivars on the *GAI*-estimation was assessed by comparing the MAEs of the models with regard to the calibration- and the evaluation dataset. Additionally, an ANOVA was performed to test whether there is a significant difference between the two models or rather a significant effect of the cultivars in the “extended” model.

**Table 3:** Characteristics of the cultivars Piko and Solehio (May 9, 2017; sowing density 400 K m^-2^).

Characteristic	Piko	Solehio
Mean Angle (°)	63.5	72.5
Stem Dry Matter/Leaf Dry Matter	1.21	2.18
Specific Leaf Area (g m^-2^)	185.94	171.95
Leaf Nitrogen Concentration (%)	4.71	4.4

For the examination of the models during the senescence we built on the approach of Serrano et al. (2000) of introducing an empirical green fraction factor. However, in contrast to the biomass-based approach of Serrano et al. (2000), a chlorophyll-driven approach was chosen. The methodology is based on SPAD-measurements which are converted into a “canopy greenness”-factor. Required data for this approach were collected by measurements with a chlorophyll meter (SPAD-502, Konica Minolta) in the growing season 2016/17 during the phase from maximal *GAI* until harvest on 13 dates (June 20, June 24, June 28, July 2, July 5, July 8, July 11, July 14, July 17, July 20, July 23, July 26, and July 30) in all leaf layers (10 leaves per layer). Used plant material was a subset of a large genotype trial (eight genotypes: Piko, Dekan, Hybery, Jafet, Biscay, SUR99820, Brilliant, and Lambriego Inia, in three replications). Crop management included 220 kg N ha^-1^ (N_min_ in early spring subtracted) and application of herbicides as well as pesticides. Tested plots were spread over a large area with much variation in soil properties, which resulted in an increased variation regarding the canopy greenness during senescence. Different leaf layers make up for different shares of the total canopy area. To account for this effect, 20 shoots of each genotype were sampled, fractionated into leaf layers, stem, and spike and the green area was determined as described before.

Multiple authors showed a nonlinear relationship between SPAD-measurements and chlorophyll concentrations (Markwell et al., 1995; Uddling et al., 2007; Ling et al, 2011). To get a closer link to the physiological base of “greenness”, we transformed SPAD-readings to chlorophyll concentrations in g m^-2^ (per unit leaf area), using the equation from Uddling et al. (2007).

Chlorophyll concentrations of each leaf layer were multiplied by its fraction of the overall canopy leaf area and the sum of these weighted concentrations is the average chlorophyll concentration of the canopy in g m^-2^.

Weibull curves were fitted on single plot level to the relationship between chlorophyll concentration and thermal time. Each value of a plot was reduced by the minimum value of its fit and afterwards divided by its maximum value, resulting in the parameter “measured canopy greenness” (ranges between one at maximal *GAI* and 0 when leaves are clearly senesced.

For the evaluation of the *GAI*-models during the senescence, all predicted values after June 19 (start of senescence) were divided by the value on June 19 to get the “predicted canopy greenness”. These predictions were hence correlated to the measured canopy greenness and the goodness of fit was assessed *via* MAE and R².

In a final step, the informative value of the calibrated models was tested by their application. This had two objectives: The evaluation of the whole season *GAI*-predictions with the different models and the illustration of the potential and suitability of the resulting *GAI*-information for agricultural research and commercial crop production.

For this purpose, we refer to a very simple but common method of correlating VI-measurements with crop characteristics (Watson et al., 1963; Pinter et al., 1981; Tucker et al., 1981; Bartholome, 1988; Rasmussen, 1992; Serrano et al., 2000). In these studies the spectral measurements were summarized in a VI, most commonly the NDVI, and, either on single dates or time-integrated over multiple dates, correlated with crop yield or final biomass. In this context, the VI represents the duration and intensity of the photosynthetic capacity of the canopy (Serrano et al., 2000) and it has been proven that the correlation of the parameters can be increased by a good performing VI (Tucker et al., 1981; Serrano et al., 2000). It seems therefore suitable to test our *GAI*-models with this approach.

Sequoia data from the 14 flights in the season 2017/18 from Trial C was hence used to calculate *GAI*-courses on plot level through the whole season with the different tested *GAI*-models. Between the dates, the *GAI* was linearly interpolated. Subsequently, the green area duration was calculated by integrating the *GAI* over the whole season. Then, the proportion of variance explained of the final biomass was examined. Furthermore, the development of the variance explained during season was considered, hence whether and how efficient the *GAI*-models convert additional multispectral-data to agronomic reasonable information.

## Results

### Linear Regression Models

None of the single bands performs convincingly (MAE_evaluation_ = 1.41–2.64 m^2^ m^-2^, **Table S2**). Combining the bands increases the performance considerably, especially if interactions between the bands are allowed (16 different terms, MAE_evaluation_ = 0.99 m^2^ m^-2^, **Table S2**). The single ratio-models perform noticeably better than the single band-models (MAE_evaluation_ = 0.55–0.81 m^2^ m^-2^, **Table 4**). The combination of different ratios provides considerably better results than the single ratios, with a lower MAE_calibration_ if interactions are allowed, but with a better performance at evaluation if not (**Table 4**). It is noteworthy that the increase of the predictive error from calibration to evaluation is considerably lower for most of the Simple Ratio approaches (on average 61%, **Table 4**), than for the single band-models (on average 80%, **Table S2**) and that the increase of the single-band model with interaction is the highest (230%).

**Table 4:** Measurement of model performance for VI-based *GAI*-prediction [m^2^ m^-2^] in calibration and evaluation with ratios of reflections as predictors and the equation for the calibrated *GAI*-models.

Linear model	MAE_calibration_ (RMSE_calibration_)	MAE_evaluation_ (RMSE_evaluation_)	Equation	
NIR/RE	0.44 (0.60)	0.55 (0.74)	-5.498 + 4.64 · NIR/RE	(1)
NIR/Red	0.45 (0.69)	0.81 (1.27)	-0.2066 + 0.1984 · NIR/Red	(2)
NIR/Green	0.36 (0.56)	0.64 (0.98)	-1.023 + 0.499 · NIR/Green	(3)
NIR/RE + NIR/Red + NIR/Green (VIQUO)	0.35 (0.53)	0.45 (0.71)	-2.829243 + 1.814068 · NIR/RE - 0.004532 · NIR/Red + 0.321576 · NIR/Green	(4)
NIR/RE × NIR/Red × NIR/Green	0.31 (0.51)	0.46 (0.72)	0.23558 - 0.72441 · NIR/RE + 0.11783 · NIR/Red - 0.02023 · NIR/Green + 0.01313 · NIR/Red · NIR/Green + 0.10864 · NIR/Red · NIR/RE + 0.28252 · NIR/RE · NIR/Green - 0.01195 · NIR/RE · NIR/Green · NIR/Red	(5)
NDVI_exp_	0.42 (0.66)	0.69 (1.04)	0.00197 · exp(8.42847 · ((NIR – Red)/(NIR + Red))	(6)
**mean**	**0.39 (0.59)**	**0.58 (0.89)**		

MAEs are colored dark grey if they are higher than the mean and white if equal or lower.

In summary, the combination of NIR/Green, NIR/Red, and NIR/RE without interaction convinces the most (MAE_evaluation_ = 0.45 m^2^ m^-2^ and RMSE_evaluation_ = 0.71 m^2^ m^-2^, **Table 4**), followed by the simple ratio of NIR/RE (MAE_evaluation_ = 0.55 m^2^ m^-2^ and RMSE_evaluation_ = 0.74 m^2^ m^-2^, **Table 4**).

These two were hence selected for further evaluation, together with the NDVI_exp_ as the most common VI. The VI combining all spectral bands provided by the Sequoia camera as NIR-based quotients was named VIQUO. The equations for the calibrated models are given in **Table 4**.

### Advanced Predictive Models

On average, the advanced predictive models perform better than the VI-models, in terms of raw reflections, reflectance ratios, in the calibration and the evaluation (**Tables 4**, **5**, **S2** and **S3**). While the raw data models provide similar MAEs in terms of calibration, their MAEs in the evaluation are at least 111% higher (**Tables 5** and **S3**).

**Table 5:** Measurement of model performance for *GAI*-prediction [m^2^ m^-2^] with advanced predictive models in calibration and evaluation with ratios of reflections as predictors.

Advanced Predictive Models	MAE_calibration_ (RMSE_calibration_)	MAE_evaluation_ (RMSE_evaluation_)	Equation	
Partial Least Squares	0.36 (0.55)	0.47 (0.76)	…	(7)
Support Vector Machine (linear Kernel)	0.36 (0.54)	0.44 (0.71)	…	(8)
Support Vector Machine (radial Kernel)	0.32 (0.51)	0.44 (0.69)	…	(9)
K Nearest Neighbor	0.33 (0.54)	0.47 (0.72)	…	(10)
Multivariate Adaptive Regression Spline	0.32 (0.51)	0.44 (0.70)	…	(11)
Boosted Trees	0.29 (0.47)	0.52 (0.82)	…	(12)
**mean**	**0.33 (0.52)**	**0.46 (0.73)**		

MAEs are colored dark grey if they are higher than the mean and white if equal or lower.

Focusing on the ratio-based models, it is noticeably that the best predictive models in calibration are the worst in the evaluation (e.g. Boosted Trees with an increase of predictive error of 79%, **Table 5**). The best models in the evaluation are the SVMs with linear respectively radial Kernel and the Multivariate Adaptive Regression Spline (MAE_evaluation_ = 0.44 m² m^-2^). Due to its relative simplicity, the SVM with linear Kernel (SVM, MAE_evaluation_ = 0.44 m² m^-2^ and RMSE_evaluation_ = 0.71 m m^-2^) is chosen to represent advanced predictive modeling methods for further investigation and comparison with the VI-models.

### Further Evaluation of GAI-Models

**Figure 2:**
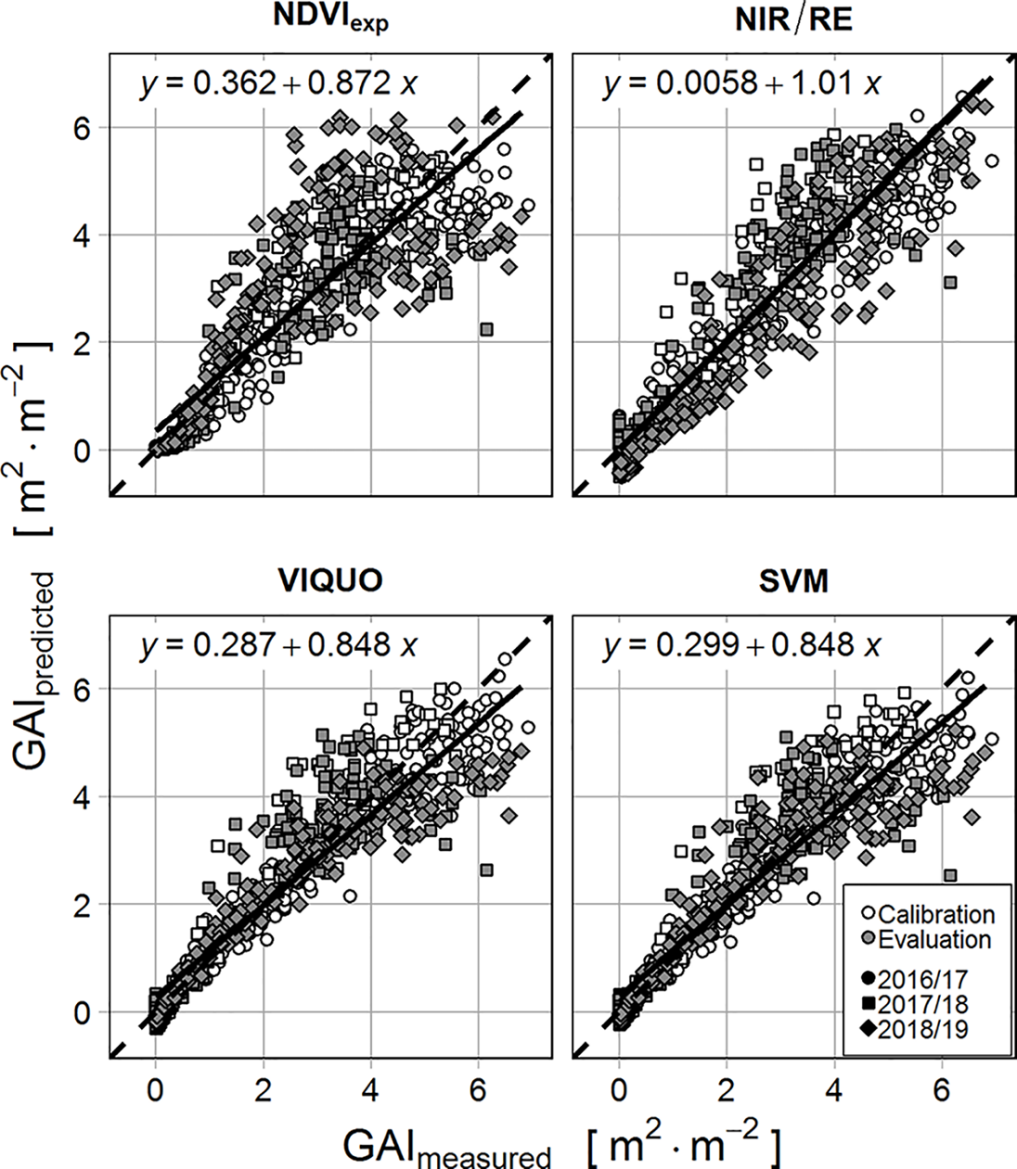
Correlation of measured and predicted *GAI* for calibration (white) and evaluation dataset (grey) for the different VIs and equation of the linear regression of measured vs. predicted values of the evaluation dataset (different shapes illustrate the three sampling seasons).

Comparing the performance of the selected VI-models with the SVM *via* their MAEs (**Tables 4** and **5**), it can be stated that the SVM performs considerably better than the NDVI_exp_ and the NIR/RE, both in terms of calibration and evaluation, whether the VIQUO has a lower MAE_calibration_ as the SVM and its MAE_evaluation_ is only slightly higher. The increase of predictive error from calibration to evaluation is relatively high with the NDVI_exp_-model (64%, **Table 4**), but nearly the same with the SVM-, the NIR/RE- and the VIQUO-model (22–29%, **Tables 4** and **5**). However, while the predictive error of SVM and VIQUO is increasing at high *GAI*-values, the predictions of NIR/RE meets the high *GAI*s well, but its predictions at low *GAI*-values scatter strongly, producing for the most part negative predictions (**Figure 2**). Looking at **Figure 2**, it is notably that several points in the high *GAI*-range in the evaluation are not met by any of the tested models. These data were obtained during the last sampling date in 2018, after a long period of drought. It is hence probable that they are already affected by senescence, which is not adequate depicted in destructive measurements.

**Figure 3:**
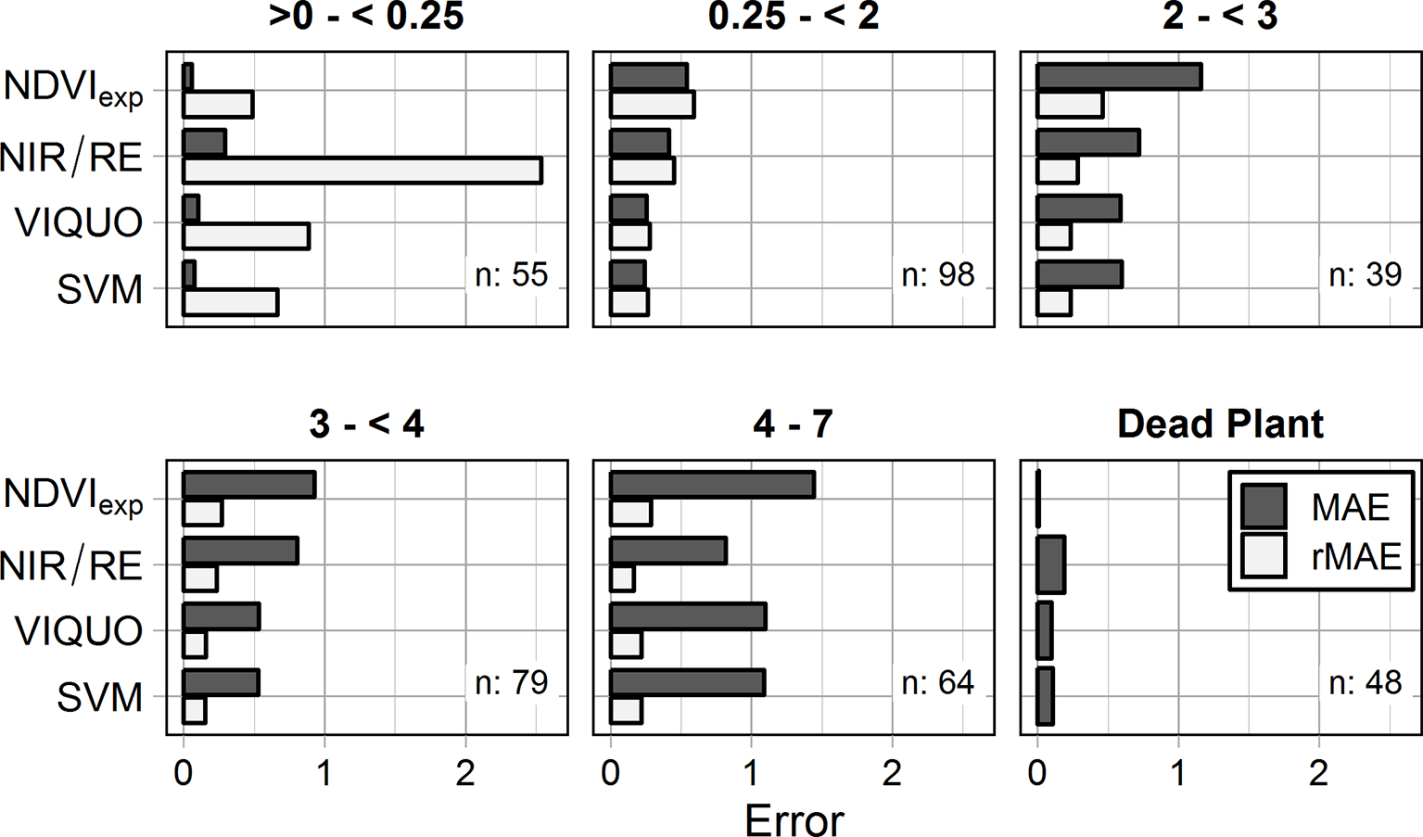
Mean absolute errors (MAE [m^2^ m^-2^]) and relative Mean absolute errors (rMAE [-]) of the different VIs for the evaluation dataset, calculated individually for the different *GAI*-classes (n: sample size of the considered *GAI*-class). rMAEs of the class dead plant cannot be calculated due to division by zero.

**Figure 3** allows a closer view to the topic of varying model-sensitivity through the season: With regard to the MAE, none of the examined VIs has considerable problems in predicting low *GAI*s (> 0 – < 0.25 & Dead Plants), with NIR/RE having the highest predictive error (MAE = 0.29 m^2^ m^-2^). Due to the exponential term, the NDVI_exp_-model predicts very well in these classes, but it has considerable problems depicting high *GAI*-values, with the highest MAEs in every other *GAI*-class (**Figure 3**) and a massive scattering when predicting *GAI*s > 2 (**Figure 2**). SVM and VIQUO perform well at the low values and are very sensitive to medium-range *GAI*s, with only small differences between the two models. However, a saturation effect is visible at *GAI*s > 5. In contrast, while the NIR/RE-model is the worst in predicting low *GAI*s (0–0.25 & Dead Plants) and only slightly better at medium *GAI*s (< 2, 2–3 and 3–4), it shows the best results when depicting *GAI*s above four (MAE = 0.81 m^2^ m^-2^, rMAE = 16%) and is the only tested approach with no saturation effects at the highest measured *GAI* values.

For the assessment of cultivar-specific effects, the linear NIR/RE-model was chosen (due to the low number of predictors and the concern to not inflate the number of interactions between cultivars and predictors). No significant difference (p = 0.05) between the model with- and without the interaction between reflectance and cultivar information was determined by means of ANOVA and the MAEs of the two models in calibration and evaluation differ only slightly (without interaction: MAE_calibration_ = 0.40 m^2^ m^-2^, MAE_evaluation_ = 0.55 m^2^ m^-2^, with interaction: MAE_calibration_ = 0.39 m^2^ m^-2^, MAE_evaluation_ = 0.54 m^2^ m^-2^).

#### Senescence

**Figure 4:**
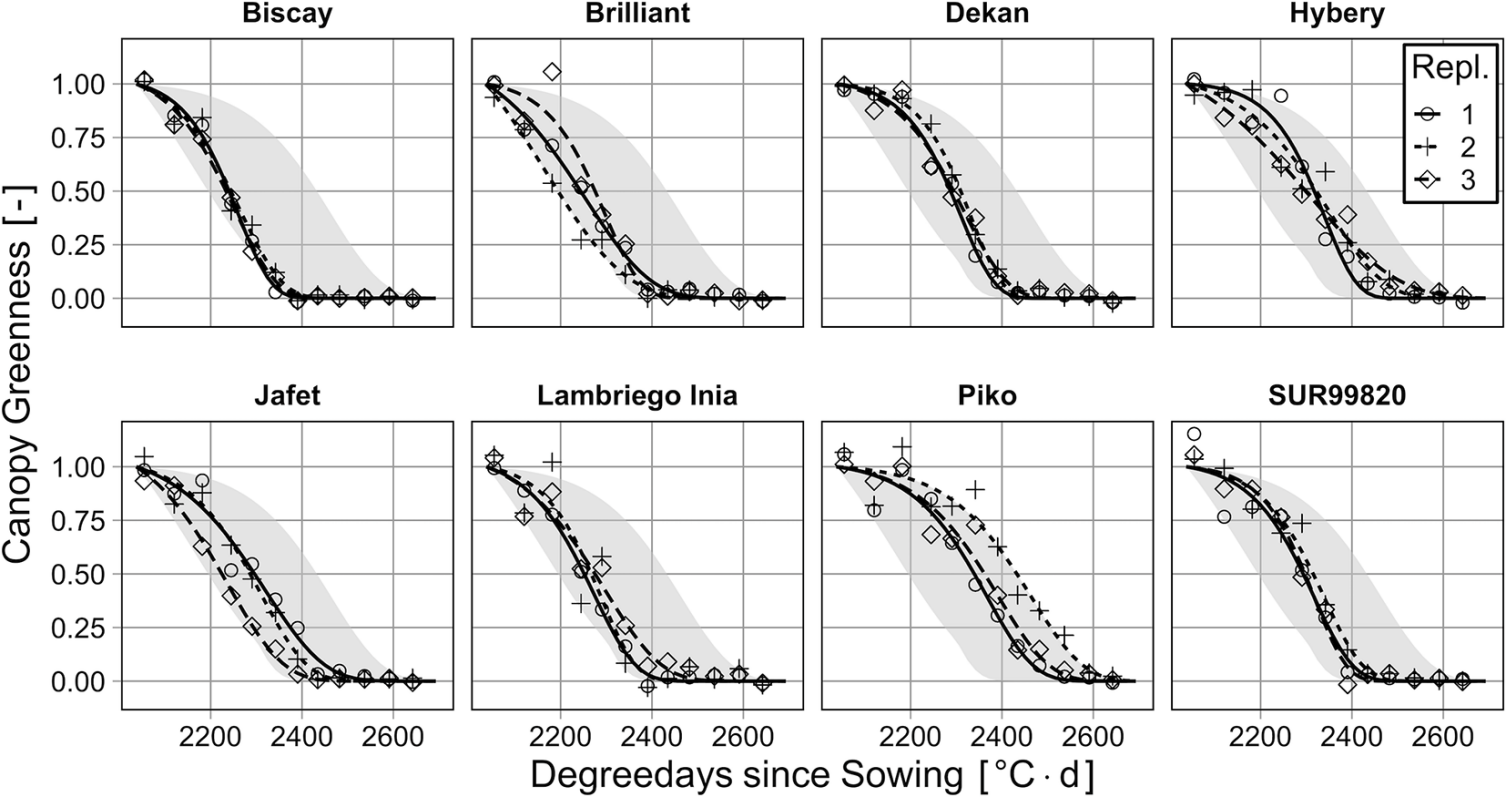
Canopy greenness for eight genotypes, grouped by replication (Repl.), in relation to degree days since sowing. The grey ribbon indicates the range of all genotypes to facilitate their classification.

Transforming the SPAD-time series to canopy greenness enables the quantification of the process of senescence (**Figure 4**). Canopy greenness varies in a large range (~300 °C d, ~15 d), due to variation of genotype and soil properties, and enables a robust model evaluation during senescence.

**Figure 5:**
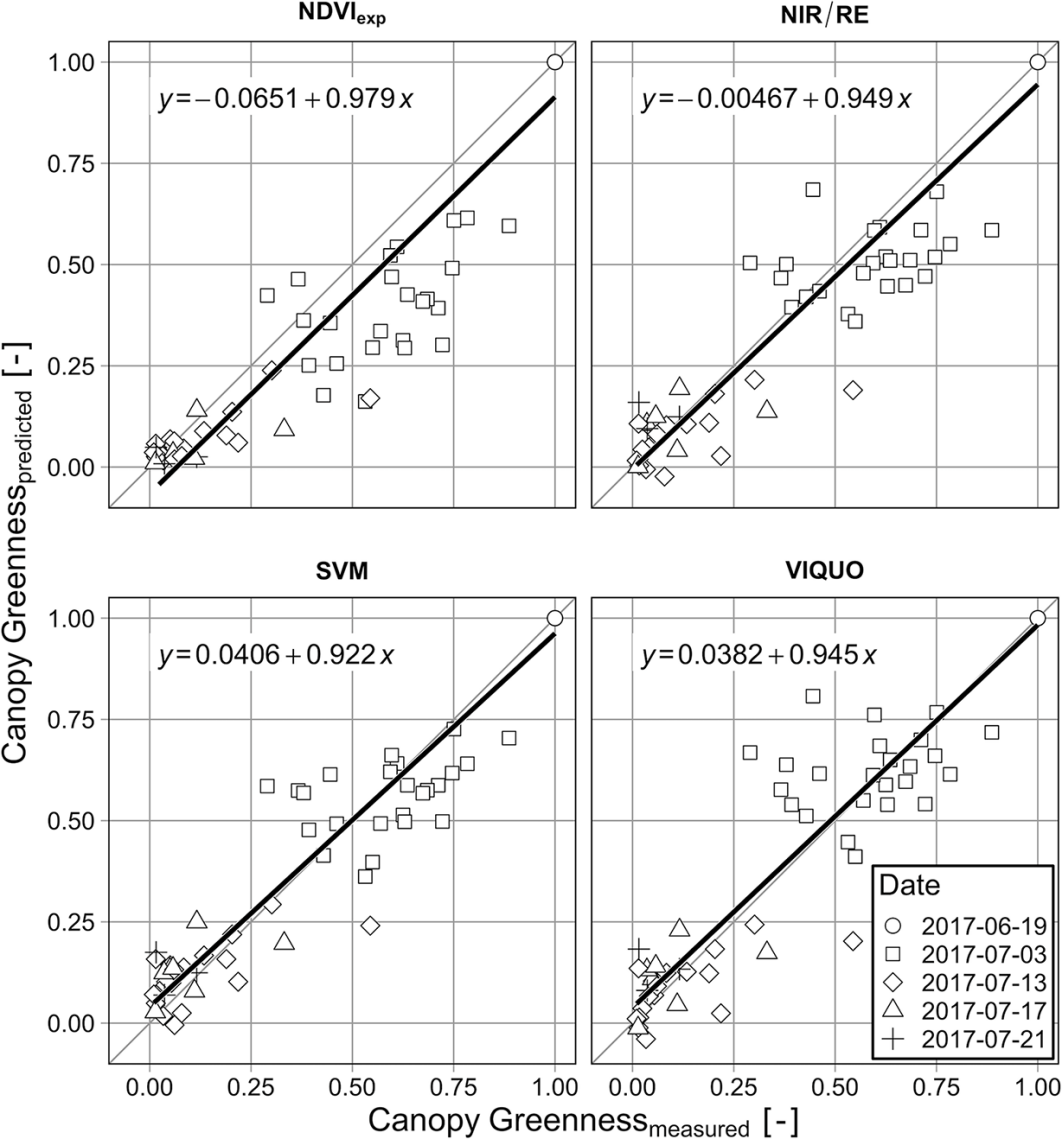
Evaluation of selected *GAI*-models during the senescence. Data from June 19 is excluded from the calculation of the RMSE because of the self-explanatory perfect fit.

The relationship between measured values of canopy greenness (SPAD-meter) and predicted values (multispectral) by the tested models for *GAI*-prediction is quite close (**Figure 5**). Regarding MAE and R^2^, the model with NDVI_exp_ is the worst-performing one (MAE = 0.13 m^2^ m^-2^; R^2^ = 0.91), followed by VIQUO (MAE = 0.10 m^2^ m^-2^; R^2^ = 0.92), NIR/RE (MAE = 0.10 m^2^ m^-2^; R^2^ = 0.93), and SVM (MAE = 0.09 m^2^ m^-2^; R^2^^=^ 0.94) as the best performing model. Especially the predictions of the VIQUO- and the SVM-calibration are nearly unbiased.

#### Suitability for Agricultural Research and Commercial Crop Production

**Figure 6:**
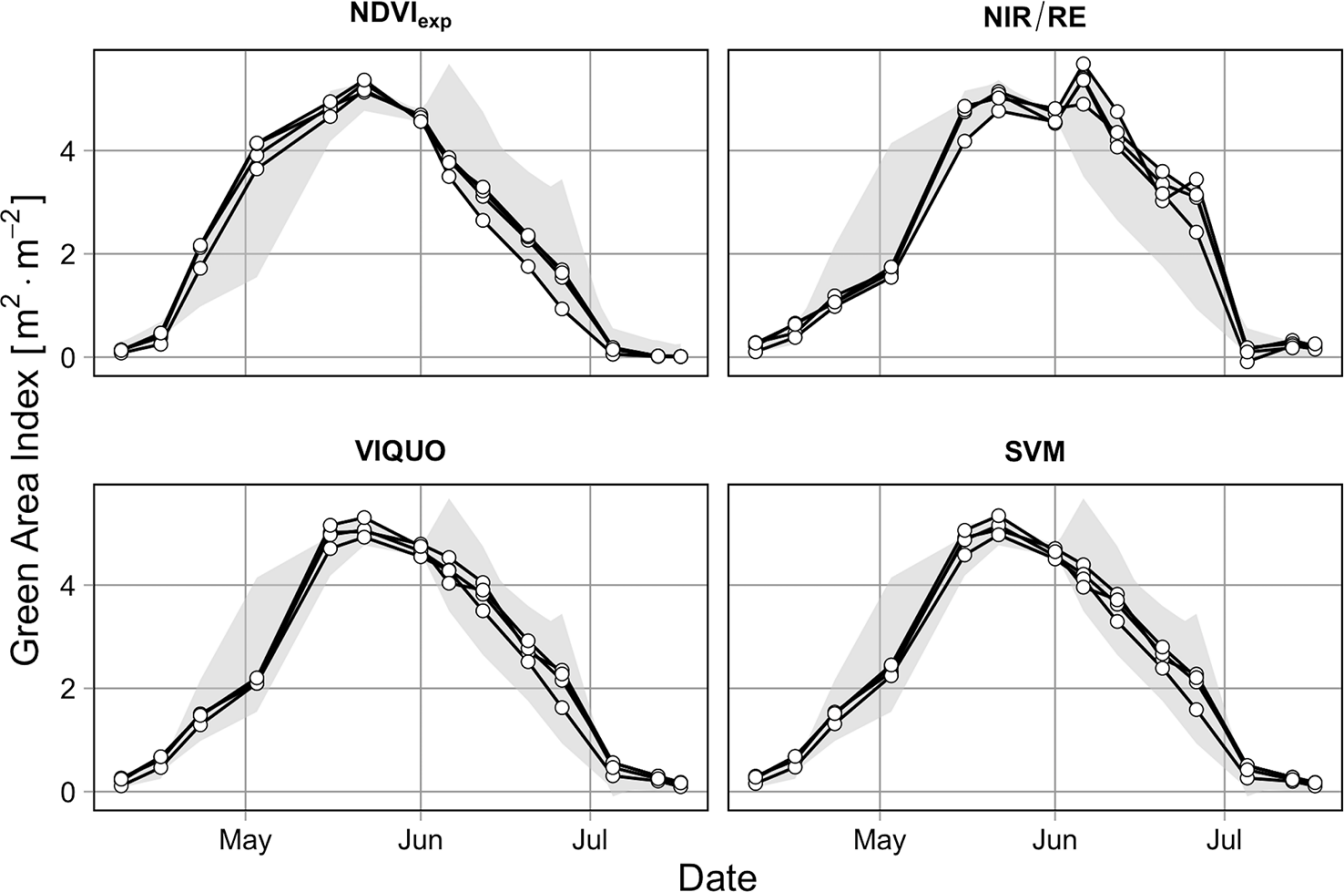
Seasonal course of predicted *GAI*s by different models in the growing season 2017/2018 on four different plots of Trial C (cultivar Solehio, 220 kg N ha^-1^). Grey ribbons indicate the range of all models.

**Figure 7:**
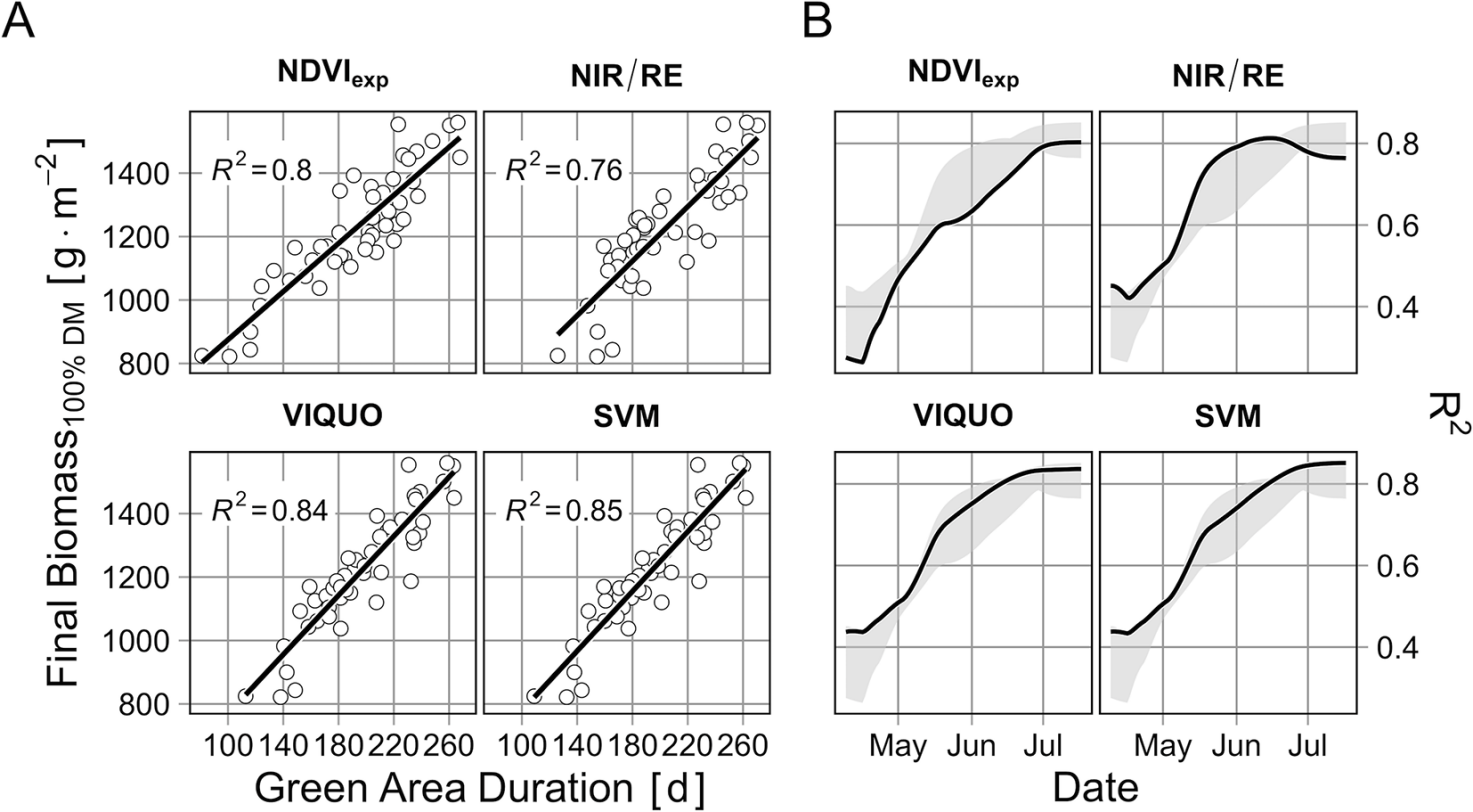
**(A)** Correlation of total green area duration and final biomass **(B)** the R^2^ of the relationship between green area duration (calculated from sowing to date *x*) and final biomass, with different models applied for the prediction of *GAI*. Final date in subfigure B corresponds to subfigure A.

The application of the selected models to the multispectral data from Trial C in 2017/18 reveals that the different *GAI-*models provide in part considerably differing results (**Figure 6**). While the *GAI*-curves of the NDVI_exp_-, the SVM- and the VIQUO-model are running very smooth and even through the season, the NIR/RE-model has problems during senescence: at two flight dates, the *GAI* seems to increase again at some plots and at the third-last date negative *GAI*s are predicted. Apart from these three “problematic” dates with regard to the NIR/RE, the *GAI*-curves of the SVM, the VIQUO, and the NIR/RE are similar, whereas the NDVI_exp_ predicts a faster *GAI*-increase from April to May and an earlier decrease from June to July, reaching a *GAI* of 0 m^2^ m^-2^ already at the first date in July. This results in notably lower NDVI_exp_-estimated green area durations for some plots (**Figure 7A**). When comparing the green area durations with the final biomasses, the correlation achieved by the NDVI_exp_-predictions is nevertheless notably better than those of the NIR/RE-model. This is attributable to the instability of the NIR/RE-model during senescence, as the explained variation decreases notably in this period (**Figure 7B**). This characteristic of the R²-curve is unique, as the explanatory power of the other *GAI*-models increases when more data is provided (**Figure 7B**). It is only due to this, that the final R² of the NIR/RE-predictions is lower than the one of the NDVI_exp_-predictions, as during the rest of the season, the NDVI_exp_-based green area duration-predictions have the lowest informative value regarding the final biomass and as the NIR/RE-predictions between May and mid of June are even the best (**Figure 7B**). It is worth mentioning that in the early May the four *GAI*-models have approximately the same informative value, explaining about 50% of the final biomass variation. The R²-curves of VIQUO and SVM increase constantly and almost equally through the season and explain finally the highest proportion of final biomass variation (> 80%, **Figure 7B**).

## Discussion

Before discussing different *GAI*-models or possible areas of application, it is necessary to consider the applicability of the Sequoia camera. Other studies using different UAV-based sensors in general (Duan et al., 2017; Zhou et al., 2017; Condorelli et al., 2018; Kanning et al., 2018), predecessors of the Sequoia sensor (Verger et al., 2014; Haghighattalab et al., 2016; Nebiker et al., 2016) and in particular the Sequoia sensor (Condorelli et al., 2018; Tunca et al., 2018) showed promising results in terms of comparability with ground-based multispectral sensors and their implication to generate information about crop characteristics. Our results match to these findings; the Sequoia camera provides reliable and sufficiently accurate data for crop monitoring purposes on plot level in terms of a scientific context. This should be transferable in the context of commercial crop production as well.

### Whole-Season Applicability

A whole season- and cultivar-transferable approach for *GAI*-prediction is preferable to specific models for several growth stages, concerning simplicity in data processing and communicability, both affecting transferability into practice. In our data, no significant cultivar-specific effect can be identified. When it comes to *GAI*-prediction, established calibration methods exist until the *GAI*-maximum is reached. But problems arise when the sensitivity of *GAI*-models is tested after the start of the senescence, as the accurate identification of the actual *GAI* is complicated or not possible at all due to the inhomogeneous senescence of the plant organs and the gradual degradation of leaf chlorophyll. Problems in estimating winter wheat *GAI* during senescence based on multispectral data have also been reported by other studies (Boegh et al., 2002; Haboudane et al., 2004; Lelong et al., 2008; Richter et al., 2012). They assumed that the decreasing performance of their calibrated models originates not from the informative value of the multispectral data but in the validity of the ground truth *GAI*-measurements. The method introduced in this study, enables the validation of our *GAI*-models during senescence and hence the confirmation of these considerations. It could thus be shown that the *GAI*-estimations of the tested models were valid and that the models, being calibrated without any senescence data (except dead plants), can provide reasonable *GAI*-predictions for this part of the season as well.

The quality of the whole-season *GAI*-predictions was tested further *via* the calculation of green area duration and the correlation with the final biomass. Hereby, 80% of the variation in the final biomass is explained by the NDVI_exp_-model, which fits to the results of Wiegand et al. (1992) and Tucker et al. (1981) with handheld devices. The new *GAI*-models VIQUO and SVM explain even more of the observed variance (up to 85%).

### Evaluation of GAI-Models

The best model to derive GAI-information should provide reliable and precise *GAI*-information as simple as possible. In terms of both, model complexity and communicability, the models can be set in the same order: The application of raw bands is the simplest approach, followed by the traditional two-band ratios. The newly introduced VIQUO-model, based on all four bands the Sequoia camera provides, follows shortly afterwards and the advanced predictive models are for sure the most complex and the most difficult to communicate. Therefore, the central question to be answered is, if the increasing complexity is worth it.

Raw reflection driven models are clearly not sufficient to predict *GAI* adequately, regardless if they are integrated in an advanced predictive model or not. The increase in MAE from calibration to evaluation indicates instability in the relationship of single bands to *GAI* between different sampling dates. Problems in predicting crop characteristics with raw reflections under varying irradiance conditions were found by Tucker et al. (1981) and Verger et al. (2014) as well, both demonstrating at the same time that this problem can be solved by using ratios instead.

When applying a two- or more-band approach, the questions of band selection and their combination arise. Many studies have worked on the issue which bands are essential to predict crop characteristics, either based on *a priori* knowledge about plant reflection characteristics (Baret and Guyot, 1991; Haboudane et al., 2004) or by recording large number of wavelengths with hyperspectral sensors and testing all possible combinations (e.g. Thenkabail et al., 2000; Hansen and Schjoerring, 2003). Based on the finding that already simple ratios (as NIR/RE) provide relatively low MAEs and on the attempt to keep the *GAI*-models as simple as possible, we restricted our analysis on two-band simple ratios, the combination of all possible simple ratios in the VIQUO and the classical NDVI (with exponential fit), and did not test different band combination approaches. In accordance to the findings of other studies (Serrano et al, 2000; Haboudane et al., 2004; Pinty et al., 2009; Viña et al., 2011) it was shown that the NDVI_exp_ is insensitive in dense canopies (here: *GAI* > 2 m^2^ m^-2^). The NIR/RE, as best Simple Ratio, is superior in predicting high *GAI*s. This result is in accordance with many studies (e.g. Delegido et al.,2013; Zhou et al., 2017) that the RE-band provides information even at high canopy densities. However, the model performs inaccurately at low *GAI*s and the NIR/RE-calculated *GAI*-curves are not stable through the season. The latter could be a result of both, of lower sensitivity of NIR/RE in the phase of senescence or of technical problems of the RE-band (the RE-band is the narrowest of the used bands, which could result in instabilities in the measurements, for example at low irradiation).

The VIQUO- and the SVM-model can compensate the temporarily low sensitivity of the RE-band and provide stable *GAI*-predictions over the whole season. A stabilization of crop-characteristic estimations with regard to differing irradiance conditions by adding additional bands to the traditional two-band approach has been reported by Mistele and Schmidhalter (2010) as well. Other studies describe a decreasing saturation effect and an increase of sensitivity obtained through additional bands (Haboudane et al., 2004; Delegido et al., 2013). Haboudane et al. (2004) increased the sensitivity of their model to *GAI*-values > 4 m^2^ m^-2^ by adding a green-band to their NIR-Red-model and achieved a whole season RMSE of 0.79 – 1.28 m^2^ m^-2^. In our case, the VIQUO, including additionally the RE-band, is sensitive to *GAI*-values ≤ 5 m^2^ m^-2^ and produces even more accurate predictions (RMSE_evaluation_ = 0.72 m^2^ m^-2^). Due to a very high fraction of intercepted radiation at *GAI*-values ≥ 5, we hold that sensitivity at rather low values is more important for the model-selection. For different requirements, the NIR/RE-model with its high sensitivity at high *GAI*-values might be the adequate approach.

The advanced predictive models are able to produce lower RMSEs than the VI-models, with the SVM even surpassing the VIQUO (RMSE_evaluation_ = 0.71 m^2^ m^-2^). This improvement in *GAI*-prediction is in accordance to the findings of Hansen and Schjoerring (2003), but notably lower than that of Verrelst et al. (2015), where the advanced predictive model reduced the RMSE by nearly 30%. This could be due to their data set, as a simultaneous consideration of different crop types might require more complex model methods, or due to their evaluation approach, as they used a cross-validation instead of an independent dataset, increasing the probability of overparameterization. While there are no indications that the here presented SVM-model is overparametrized (same decrease in predictive power from calibration to evaluation than the testes Simple Ratio-models), it has the same saturation effect for *GAI*-values >5 as the VIQUO. Considering the sensitivity in the different *GAI*-classes, the SVM is predicting mostly, but not in every case, *GAI* more precise than the VIQUO. The *GAI*-curves estimated with these two different models are nearly the same and the correlation of green area duration to final biomass of the SVM-model is consequently not notably better. On this base we consider the higher complexity of the advanced predictive model as not justified.

### Transferability of the GAI-Calibration

Lastly, besides the validity through the whole season for different cultivars, the applicability of the *GAI*-calibration across seasons should be considered. In general, the transferability of purely statistic-based approaches, as the calibration here presented, might be regarded as problematic. Additionally, the use of destructive *GAI*-data restricts the size of the data set to the affordable workload and the local conditions of the respective study site (e.g. number of seasons, plots, cultivars, nitrogen levels). A concept to overcome these problems is the use of radiative transfer models, such as PROSAIL, to generate reflectance- and *LAI*-data sets for the sensor of interest (Richter et al., 2010; Verger et al., 2014). However, this approach relies on the estimation of several crop parameters (e.g. average leaf angle, dry matter content, leaf mesophyll parameter). These parameters may vary in plot trials, for example due to different cultivars and fertilization levels (as exemplarily shown above for two cultivars), and probably also on farm sites with heterogeneous crop growth. Furthermore problematic may be to depict the sensitivity of the raw bands to illumination conditions during image acquisition when generating artificially reflectance data (Verger et al., 2014) and the saturation effect when considering high *GAI*s (Richter et al., 2010; Verger et al., 2014). These effects could be examined closer in further investigations, using our data set as basis for evaluation. However, taking into account these restrictions of physically based calibration approaches, statistically-based approaches can probably be considered at least of equal standing and have shown to be stable over multiple seasons and many different measurement dates in the presented data set.

## Conclusion

The Sequoia multispectral camera was identified as an adequate instrument for multispectral data acquisition for crop monitoring.

Different models for *GAI*-estimation were presented and evaluated. For this purpose, a new approach for evaluating *GAI*-models during senescence was introduced and tested successfully.

Only two of the tested *GAI*-models can be considered as reliable and sufficiently accurate for whole-season *GAI*-prediction; the newly developed four-band VI-approach VIQUO and the advanced predictive model SVM. Both models use all four spectral bands provided by the Sequoia camera. The two-band approaches are outperformed in terms of stability and sensitivity. Only if especially sensitivity at high GAI-values is of major importance, the alternative use of the NIR/RE-model should be considered.

The VIQUO-model is recommended as best model to estimate winter wheat *GAI*, as it provides a high precision in *GAI*-prediction and is still relatively simple, thus easier to communicate and to apply than the SVM.

The strong correlation between green area duration (derived from *GAI*-predictions) and the final biomass demonstrates the high potential of the used system (in combination with appropriate calibration) for the application in agriculture research and precision farming.

## Data Availability Statement

The datasets generated for this study are available on request to the corresponding author.

## Author Contributions

JB was coordinating author and in cooperation with TR conducting the major part of measurements and writing process. RE provided sampling and base analysis for the part about senescence during his bachelor thesis. HK was supervisor of the whole process, defined central ideas of the article and corrected the manuscript

## Funding

This work was supported by the foundation Schleswig-Holsteinische Landschaft and the Federal Ministry of Education and Research (BMBF) (grant number: 031A354D). Publication fees were supported by Land Schleswig-Holstein within the funding program Open Access Publikationsfonds.

## Conflict of Interest

The authors declare that the research was conducted in the absence of any commercial or financial relationships that could be construed as a potential conflict of interest.
