# Supplementary material for: High-Throughput Prediction of Whole Season Green Area Index in Winter Wheat With an Airborne Multispectral Sensor
Source: Front Plant Sci. 2020 Feb 14;10:1798. doi: 10.3389/fpls.2019.01798 (PMC7033565; doi:10.3389/fpls.2019.01798)
Supplement: Supplementary file 1 [file Table_1.docx]

**Table S1:** Selected tuning parameters of models with differing types of predictors: raw reflections and ratios of reflections.

|  |  | **Reflections** | **Indices** |
| --- | --- | --- | --- |
| Partial Least Squares | n. components | 3 | 1 |
| Support Vector Machine (linear Kernel) | C | 0.25 | 0.025 |
| Support Vector Machine (radial Kernel) | C | 64 | 32 |
|  | sigma | 0.001 | 0.02 |
| K Nearest Neighbor | k | 15 | 79 |
| Multivariate Adaptive Regression Spline | nprune | 6 | 3 |
|  | degree | 2 | 1 |
| Boosted Trees | ntrees | 490 | 800 |
|  | int. depth | 1 | 1 |
|  | shrinkage | 0.1 | 0.01 |
