# Supplementary material for: High-Throughput Prediction of Whole Season Green Area Index in Winter Wheat With an Airborne Multispectral Sensor
Source: Front Plant Sci. 2020 Feb 14;10:1798. doi: 10.3389/fpls.2019.01798 (PMC7033565; doi:10.3389/fpls.2019.01798)
Supplement: Supplementary file 2 [file Table_2.docx]

**Table S2**: Measurement of model performance for VI-based *GAI*-prediction [m^2^ m^-2^] in calibration and evaluation with raw reflections as predictors and the equation for the calibrated *GAI*-models. MAEs are colored dark grey if they are higher than the mean and white if equal or lower.

| **Linear Model** | **MAE_calibration_ (RMSE_calibration_)** | **MAE_evaluation_ (RMSE_evaluation_)** | **Equation** |  |
| --- | --- | --- | --- | --- |
| NIR | 0.61 (0.80) | 1.48 (1.89) | -1.853 + 10.384 · NIR | (13) |
| RE | 1.11 (1.36) | 2.64 (3.25) | -3.651 + 25.929 · RE | (14) |
| Red | 1.12 (1.32) | 1.41 (1.91) | 4.255 – 34.573 · Red | (15) |
| Green | 1.29 (1.52) | 1.48 (1.89) | 6.179 – 57.117 · Green | (16) |
| NIR + RE + Red + Green | 0.36 (0.54) | 0.84 (1.16) | 1.038 + 11.109 · NIR – 3.229 · RE + 17.736 · Red – 49.521 · Green | (17) |
| NIR x RE x Red x Green | 0.30 (0.50) | 0.99 (1.85) | -1.719 + 36.033 · NIR – 5.189 · RE + 10.994 · Red – 11.158 · Green – 68.284 · NIR · RE – 132.262 · NIR · Red – 318.731 · NIR · Green – 50.841 · RE · Red + 78.525 · RE · Green + 208.629 · Red · Green + 529.612 · Red · RE · NIR – 425.743 · Red · RE · Green + 565.758 · Red · NIR · Green + 793.791 · RE · NIR · Green – 2766.404 · Red · RE · NIR · Green | (18) |
| **mean** | **0.80 (1.00)** | **1.47 (1.99)** |  |  |
