# Supplementary material for: High-Throughput Prediction of Whole Season Green Area Index in Winter Wheat With an Airborne Multispectral Sensor
Source: Front Plant Sci. 2020 Feb 14;10:1798. doi: 10.3389/fpls.2019.01798 (PMC7033565; doi:10.3389/fpls.2019.01798)
Supplement: Supplementary file 3 [file Table_3.docx]

**Table S3**: Measurement of model performance for *GAI*-prediction [m^2^ m^-2^] with advanced predictive models in calibration and evaluation with raw reflections as predictors. MAEs are colored dark grey if they are higher than the mean and white if equal or lower.

| **Advanced Predictive Models** | **MAE_calibration_ (RMSE_calibration_)** | **MAE_evaluation_ (RMSE_evaluation_)** | **Equation** | |
| --- | --- | --- | --- | --- |
| Partial Least Squares | 0.36 (0.54) | 0.76 (1.08) | … | (19) |
| Support Vector Machine (linear Kernel) | 0.36 (0.54) | 0.84 (1.18) | … | (20) |
| Support Vector Machine (radial Kernel) | 0.34 (0.52) | 0.84 (1.14) | … | (21) |
| K Nearest Neighbor | 0.29 (0.50) | 0.97 (1.33) | … | (22) |
| Multivariate Adaptive Regression Spline | 0.32 (0.49) | 0.92 (1.32) | … | (23) |
| Boosted Trees | 0.26 (0.41) | 0.90 (1.26) | … | (24) |
| **mean** | **0.32 (0.50)** | **0.87 (1.22)** |  |  |
